# Healthcare costs at the end-of-life among immigrant and non-immigrant groups in Manitoba, Canada

**DOI:** 10.23889/ijpds.v9i5.2619

**Published:** 2024-09-18

**Authors:** Shantanu Debbarman, Julia Witt, Umut Oguzoglu, Marcelo L. Urquia

**Affiliations:** 1 Manitoba Centre for Health Policy; 2 Department of Economics, Faculty of Arts, University of Manitoba; 3 Manitoba Centre for Health Policy, Community Health Sciences, Max Rady College of Medicine, Rady Faculty of Health Sciences, University of Manitoba; 4 Dalla Lana School of Public Health, University of Toronto

**Keywords:** International Migrant, Long-term Resident, Government Assisted and Blended Visa Office-Referred Refugees

## Objectives

It is known that healthcare costs tend to increase during the last year of life. Recognizing the importance of efficient resource distribution for end-of-life care, this study compares healthcare costs incurred for migrants and long-term Manitobans and identifies the factors that impact healthcare costs during the last year of life.

## Methods

This retrospective matched-cohort study used 15 databases linked at the individual-level, including immigration records, medical claims, hospital abstracts, drug prescriptions, emergency department visits, home care, long-term care, vital statistics mortality, housing and employment/income assistance, for those who died between January 2005 and December 2022 in Manitoba. Conditional zero-inflated gamma hurdle (ZIG) and quantile regression models were used.

## Results

The average end-of-life healthcare costs for international migrants (2469) and Long-term Manitobans (2362) were CA$44,909 and CA$16,593, respectively. According to the adjusted ZIG model, international migrants had 17% higher costs. Among international migrants, Government Assisted and Blended Visa Office-Referred Refugees (GAR/BVOR) had 36% higher costs than Long-term Manitobans. Additionally, costs were higher for those without a partner (13%), receiving employment/income assistance (30%), having higher comorbidity (309% for 4+ comorbidities vs. 0 comorbidities), death at the hospital (171%), and long-term care (169%). Adjusted Quantile regression analyses revealed that only GAR/BVOR had higher costs across all levels of the cost distribution than long-term Manitobans.

## Conclusions

In the last year of life, international migrants incurred greater healthcare costs than non-immigrants. However, the differences with non-immigrants varied depending on international migrants’ characteristics.

